# Electronically Switchable Broadband Metamaterial Absorber

**DOI:** 10.1038/s41598-017-05330-z

**Published:** 2017-07-07

**Authors:** Dongju Lee, Heijun Jeong, Sungjoon Lim

**Affiliations:** School of Electrical and Electronic Engineering, Chung-Ang University, 221 Heukseok-Dong Dongjak-Gu, Seoul, 156-756 Republic of Korea

## Abstract

In this study, the novel electronically switchable broadband metamaterial absorber, using a PIN diode, is proposed. The unit cell of the absorber was designed with a Jerusalem-cross resonator and an additive ring structure, based on the FR-4 dielectric substrate. Chip resistors and PIN diodes were used to provide both a broadband characteristic and a switching capability. To satisfy the polarization insensitivity, the unit cell was designed as a symmetrical structure, including the DC bias network, electronic devices, and conductor patterns. The performance of the proposed absorber was verified using full-wave simulation and measurements. When the PIN diode was in the ON state, the proposed absorber had a 90% absorption bandwidth from 8.45–9.3 GHz. Moreover, when the PIN diode was in the OFF state, the 90% absorption bandwidth was 9.2–10.45 GHz. Therefore, the absorption band was successfully switched between the low-frequency band and the high-frequency band as the PIN diode was switched between the ON and OFF states. Furthermore, the unit cell of the proposed absorber was designed as a symmetrical structure, and its performance showed insensitivity with respect to the polarization angle.

## Introduction

Lots of effort has gone towards the reduction of the radar cross section (RCS), and the development of low-observable technologies, such as stealth technology. The metamaterial-based absorber is one such technology. The metamaterial absorber was first introduced by N. Landy^1^ in 2008 and a lot of studies have been conducted due to its various advantages^2–4^. Firstly, it has a near perfect absorptivity despite its thinness, which is unlike conventional absorbers such as ferrite^5^ and the Salisbury screen absorber^6^. Next, the absorption frequency of the metamaterial based absorber is easily controlled by artificially adjusting the effective permittivity and permeability^7^. Therefore, it can be easily designed and implemented for applications and technologies of various frequency bands, from microwaves to optical signals^8–10^. Finally, it consists of dielectric and conductor patterns that cause LC resonance, and it operates as a radar absorbing structure (RAS)^11^, not a radar absorbing material (RAM). It also operates in the form of the periodic structure array of the identical unit cell^12^. Furthermore, because of these characteristics, it can easily be expanded to the required size by designing only the unit cell.

However, the metamaterial absorber has a narrow absorbing frequency bandwidth, because its absorption is from electric and magnetic resonances. The metamaterial absorber therefore has great difficulty in coping with modern radar signals in various frequency bands. In order to overcome this problem, several researches have been conducted about the wide band, switchable, and tunable properties of the absorber. For example, an absorber using multiple resonances can be presented as a solution^13, 14^. However, it is difficult to handle the multiple resonances simultaneously, and it is still insufficient to have a wide band characteristic. On the other hand, the most common method to change the frequency band is to use electronic devices such as PIN diodes, varactor diodes, and so on^13, 15, 16^. When using electronic devices, instantaneous frequency variation is possible. The use of these electronic devices allows immediate frequency variation, but the design a separate DC bias network to operate the electrical device, which can lead to performance limitation in some cases, is inevitable. In addition to using electronic devices, the use of microelectromechanical systems (MEMS)^17^, liquid crystal^18^, and liquid metal^19^ can switch and tune the absorption frequency band. In order to design an electrically tunable metamaterial absorber, an electronic tunable device must be chosen after considering its applications, frequencies, and costs. A varactor diode is useful to continuously tune capacitance. MEMS components are useful for low power consumption and millimeter-wave bands. Tuning range and speed of liquid crystal is narrower than other components. In this work, we aim to switch between two different states rather than changing capacitance continuously. In addition, MEMS switches are too expensive to realize a periodic structure in X-band.

In this paper, a novel electronically switchable broadband metamaterial absorber is proposed using PIN diodes. The unit cell of the proposed absorber is designed as an additive ring structure, with a Jerusalem cross (JC) resonator. The additive ring structure can generate the electric field inside the unit cell, and chip resistors are placed at this location to provide a wideband characteristic. PIN diodes are used as switchable components, for the ON/OFF state. To satisfy the polarization insensitivity, the unit cell was designed as a symmetrical structure including the DC bias network, electronic devices, and conductor patterns. The proposed absorber was fabricated by using a wet etching process, and electronic devices such as PIN diodes, chip resistors, and chip inductors were attached onto the substrate, using SMT (surface mount technology). The performance of the proposed absorber was verified through full-wave simulation and measurements.

## Results

### Absorber design

**Figure 1:**
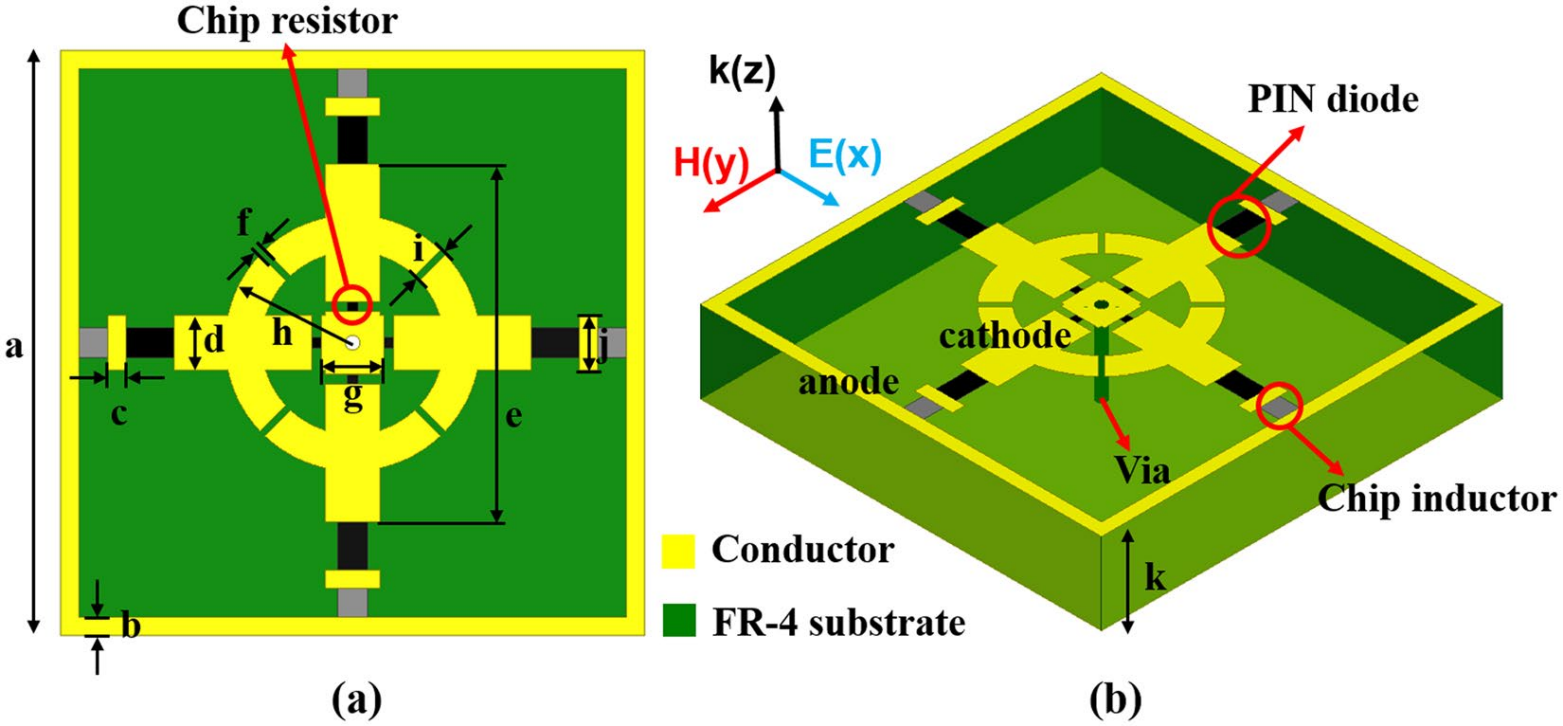
Geometry of the proposed absorber unit cell **(a)** top view, **(b)** 3-D view: *a* = 16 mm, *b* = 0.5 mm, *c* = 0.5 mm, *d* = 1.5 mm, *e* = 9.8 mm, *f* = 0.2 mm, *g* = 1.74 mm, *h* = 3.5mm, *i* = 1 mm, *j* = 1 mm, and *k* = 3.2 mm.

Figure 1 illustrates the geometry of the proposed absorber unit cell. On the top layer of the unit cell, conductor patterns are composed of the Jerusalem cross (JC) resonator, the additive ring structure, and the DC bias network. Moreover, there are four chip resistors, four PIN diode, and four chip inductors. Generally, LC resonance occurs in the JC resonator, and an electric field is generated. The additive ring structure can serve to gather the generated electric field inwards, toward the unit cell, and chip resistors are placed at this location to provide broadband operation. PIN diodes are used as switchable component, for the ON/OFF state. A separate DC bias network is required to operate the PIN diode. In this case, in order to minimize the performance limitation of the absorber due to the DC bias network, the cathode is designed as a conductor portion at the centre of the unit cell and the anode is designed as a conductor portion at the outermost part of the unit cell. The bottom layer of the unit cell is a fully covered conductor, to prevent the transmission wave. In addition, because the cathode conductor portions on the top layer and bottom layer are connected by via holes, the PIN diode can be powered through the bottom conductive layer. In the design process, to satisfy the polarization insensitivity, the unit cell is designed as both a horizontally and vertically symmetrical structure, including the DC bias network, electronic devices, and conductor patterns.

### Fabricated absorber prototype

**Figure 2:**
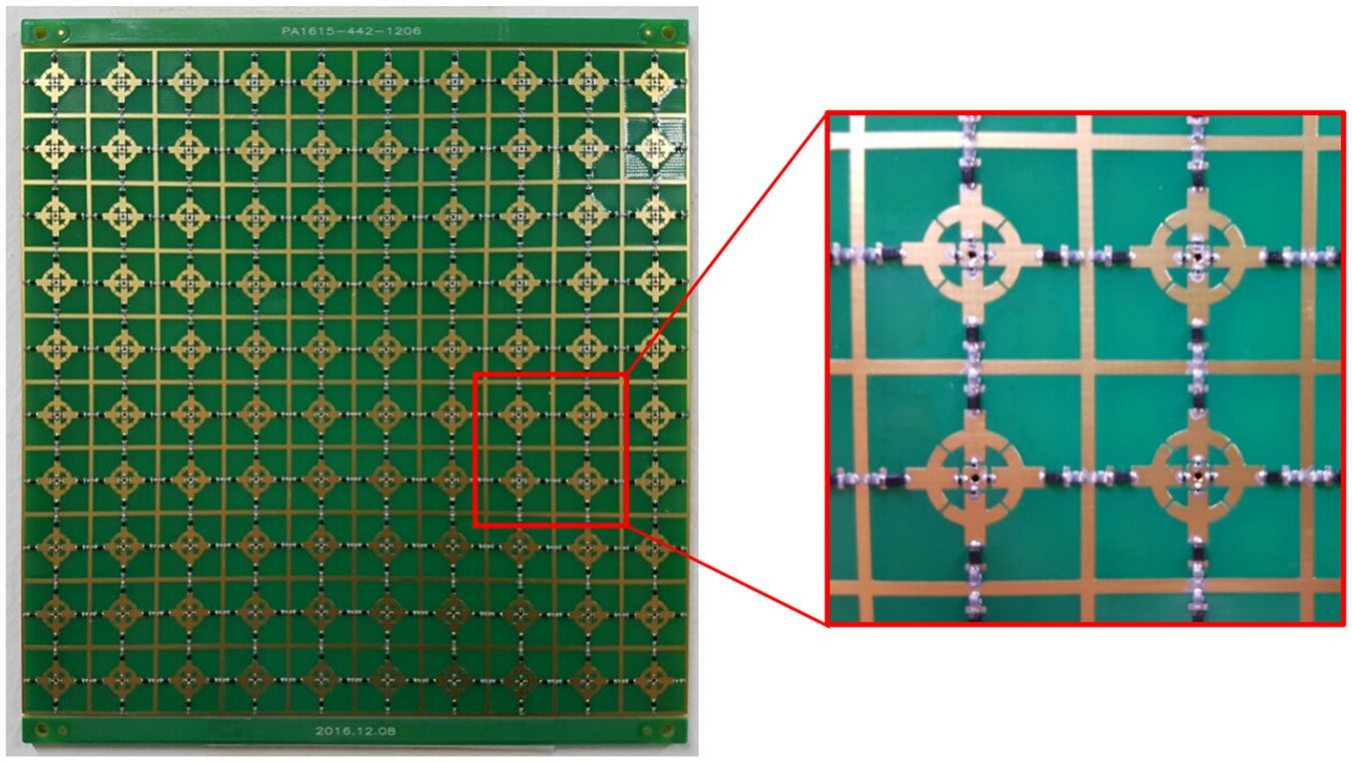
Fabricated prototype sample.

To verify the performance of the designed absorber, a prototype sample was fabricated, as shown in Fig. 2. The fabricated samples consisted of a total of 100 unit cells with a unit size of 10 × 10 and a total size of 162 × 162 mm. The FR-4 dielectric substrate was used and its relative permittivity and dielectric loss are 4.5 and 0.02, respectively. The conductor patterns on the top and bottom layers were implemented using a wet etching process. Electronic devices such as PIN diodes, chip resistors, and chip inductors were soldered on by SMT. In the fabricated prototype sample, a total of 400 chip resistors, 400 chip inductors, and 400 PIN diodes, were used. The value and the size of the chip resistors are 100 Ω and 0603 size (metric). In order to prevent the radio frequency (RF), chip inductors are used. The chip inductor used the 0402HP-3NSX_L and its value and size are 3.3 nH and 1005 size (metric). Moreover, because its self resonant frequency (SRF) is 12.8 GHz, this chip inductor is suitable for the proposed absorber, which operates in the X-band (8–12 GHz). The wires are connected to the outermost part of the top and bottom layers of the fabricated prototype, in order to supply the PIN diodes with power.

### Simulated and experimental results

**Figure 3:**
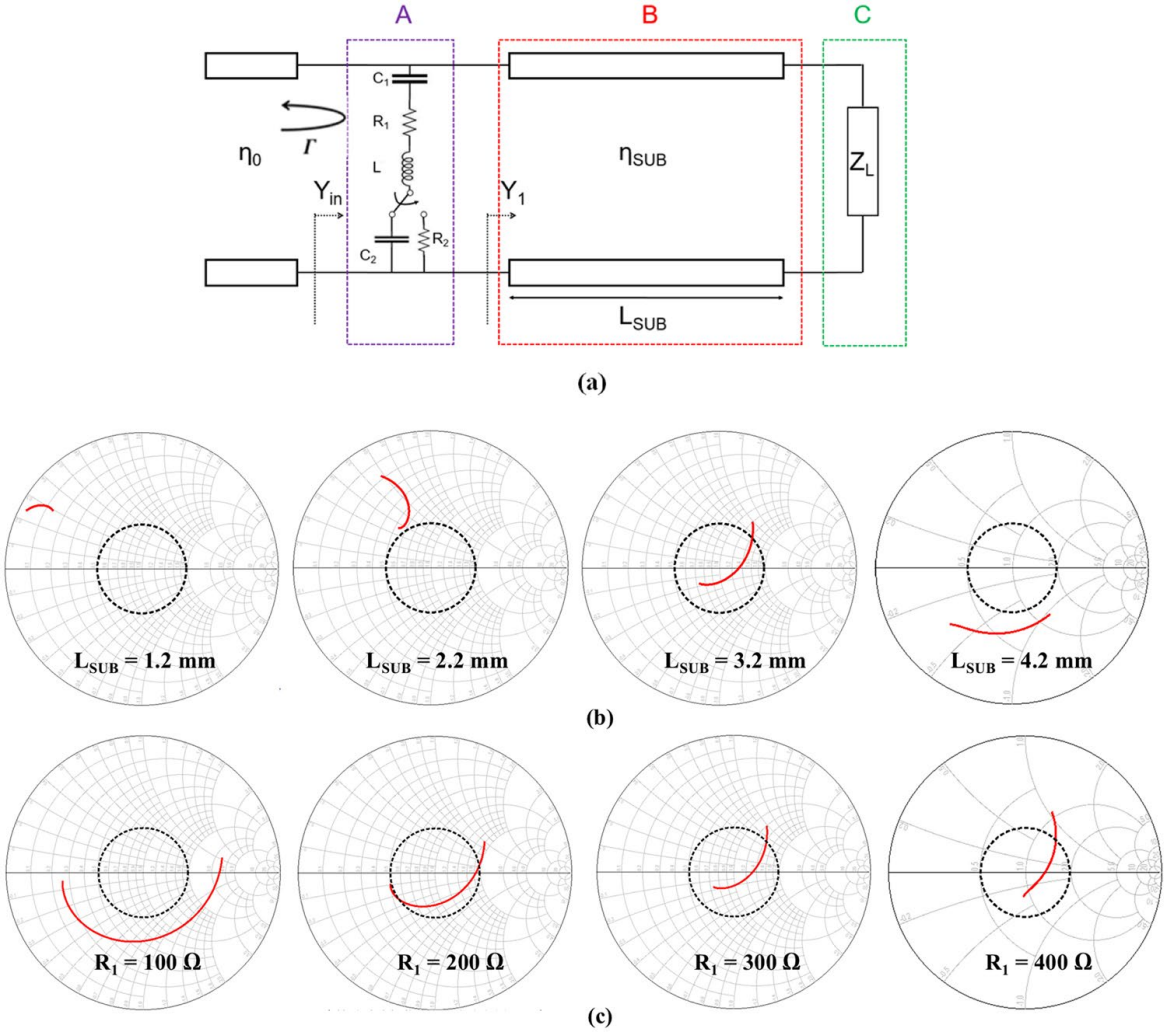
(**a**) Transmission line mode. (**b**) Input impedance traces from 8 to 12 GHz on Smith chart at different (**b**) L_SUB_ and (**c**) R_1_. The dotted line is 2:1 VSWR circle.

In this study, a SMP1340-079LF PIN diode provided by Skyworks Solutions, Inc. is used. The equivalent circuit of the PIN diode is represented by R (resistor), L (inductance), and C (capacitance) as its ON and OFF states^20^. When the PIN diode is in the OFF state, it is modelled as a series L, a parallel C_2_, and a high-value R_P_. When the diode is in the ON state, it is also modelled as an L and R_2_ in series. The component values are L = 0.45 nH, R_2_ = 5 Ω, C_2_ = 0.03 pF, and R_P_ = 5 MΩ. Consequently, the proposed absorber can be expressed as a transmission line model, consisting of three section, A, B, and C, as shown in Fig. 3(a). The first section, ‘A’, demonstrates the top layer of the proposed absorber. The conductive patterns on the top layer are represented by C_1_, R_1_, and L in series, and are switched to C_2_ and R_2_ as the PIN diodes is switched between the ON and OFF state, respectively. In the section, ‘B’, the finite length of the transmission line (L_SUB_) represents the dielectric substrate, with an intrinsic impedance of η_SUB_. Finally, section ‘C’ represents the ground (GND) plane as a load impedance of Z_L_.

The reflection coefficient is given by the flowing equation:

1 $${\rm{\Gamma }}(\omega )=\frac{{Y}_{0}-{Y}_{{in}}}{{Y}_{0}+{Y}_{{in}}},$$

where Y_0_ is the admittance of free space.

Consequently, the zero reflection coefficient is achieved when Y_in_ and Y_0_ are equal. Figure 3(b) and (c) show the input impedance of the transmission line model from 8 to 12 GHz on Smith chart. L_SUB_ and R_1_ are critical parameters for broadband impedance matching. For instance, as L_SUB_ is increased as shown in Fig. 3(b), the impedance trace is approaching to the center. In addition, when L_SUB_ is 3.2 mm, the impedance trace is inside 2:1 VSWR (voltage standing wave ratio) circle. From Fig. 3(c), the impedance trace is approaching to the center as R_1_ increases. Especially, when R_1_ is 300 Ω, the impedance trace is inside 2:1 VSWR circle.

**Figure 4:**
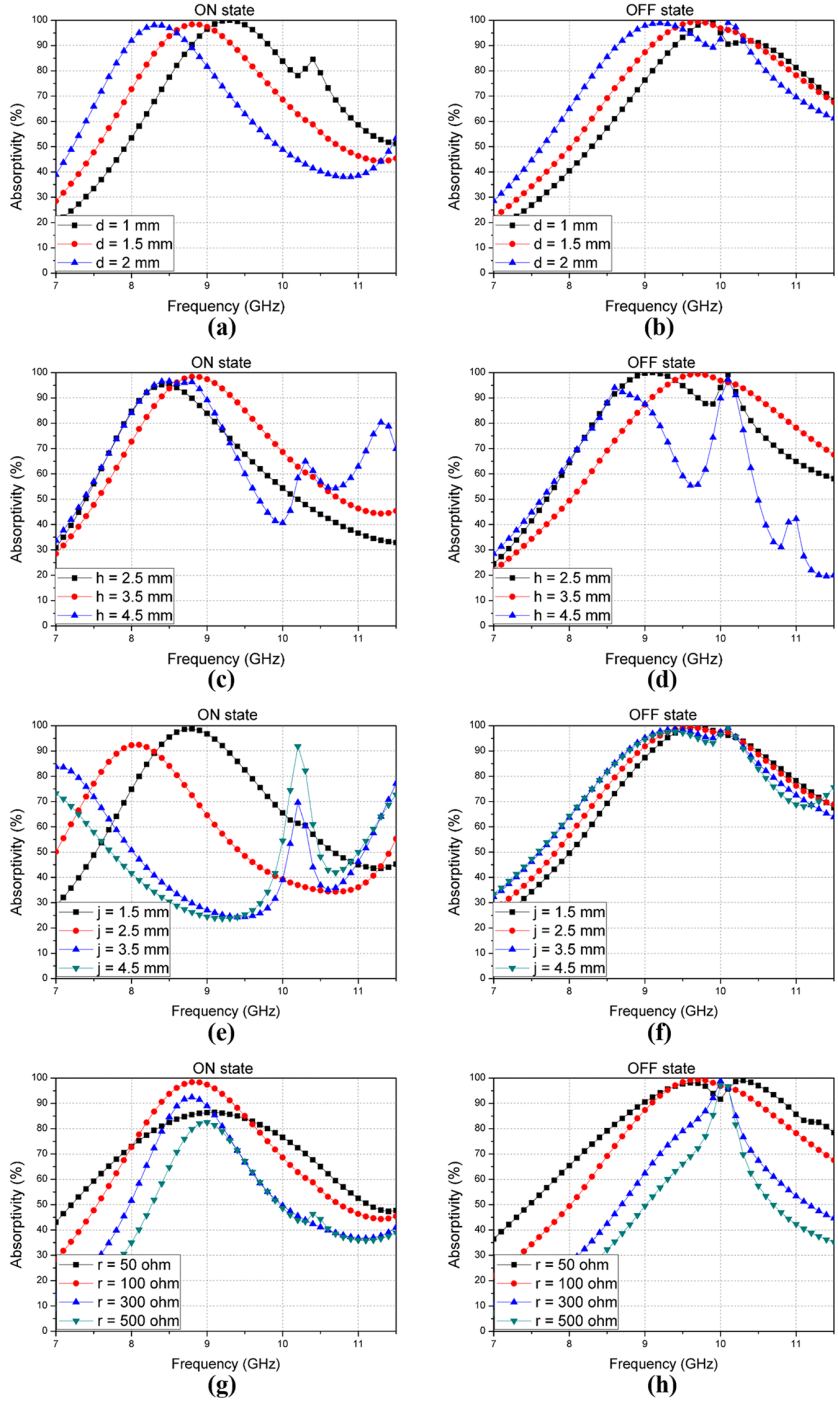
Simulated absorptivity at different values of design parameters: **(a)** ON state when value of ‘d’ varies from 1 mm–2 mm, **(b)** OFF state; **(c)** ON state when value of ‘h’ varies from 2.5 mm–4.5 mm, **(d)** OFF state; **(e)** ON state when value of ‘j’ varies from 1.5 mm–4.5 mm, **(f)** OFF state; **(g)** ON state when value of ‘r’ varies from 50 Ω–500 Ω, **(h)** OFF state.

Figure 4 shows the variation of the absorptivity with the change of the design parameters such as d, h, j, and the value of the chip resistor (r). In the design process, since the absorption frequency is switched depending on the state of the PIN diode, the design parameters satisfying both conditions are determined through full-wave simulation. Firstly, Fig. 4(a) and (b) show the variation of the absorptivity with the change of the width of JC, the width being represented by ‘d’. Regardless of the state of the PIN diode, the absorption frequency band increases as the value of ‘d’ decreases from 2 mm to 1 mm. However, when the PIN diode is in the OFF state, the absorption bandwidth is the widest and most stable at a ‘d’ of 1.5 mm. Secondly, the change of the absorptivity is shown in Fig. 4(c) and (d), when the radius ‘h’, of the additive ring structure, varies from 2.5 mm–4.5 mm. When the PIN diode is in the ON state, the absorptivity is best at an ‘h’ of 3.5 mm. Furthermore, when the PIN diode is in the OFF state, the absorption bandwidth is the widest and most stable at an ‘h’ of 3.5 mm. Next, Fig. 4(e) and (f) show the variation of the absorptivity when the width of stub ‘j’ varies from 1.5 mm–4.5 mm. In this case, when the PIN diode is in the OFF state, there are slight differences at various values of ‘j’. However, when the PIN diode is in the ON state, the absorptivity is best, and the absorbing bandwidth is the widest at a ‘j’ of 1.5 mm. Finally, Fig. 4(g) and (h) show the change of the absorptivity when the value of the chip resistors (r), ranges from 100 Ω–500 Ω. When the PIN diode is in the ON state, the absorptivity is best, and the bandwidth is widest at an ‘r’ of 100 Ω. Likewise, when the PIN diode is in the OFF state, the absorptivity is best, and the absorption bandwidth decreases as ‘r’ becomes lager. As a result, the various design parameters of the proposed absorber are determined as follows: *a* = 16 mm, *b* = 0.5 mm, *c* = 0.5 mm, *d = *1.5 mm, *e* = 9.8 mm, *f* = 0.2 mm, *g = *1.74 mm, *h* = 3.5mm, *i = *1 mm, *j = *1 mm, and *k = *3.2 mm.

**Figure 5:**
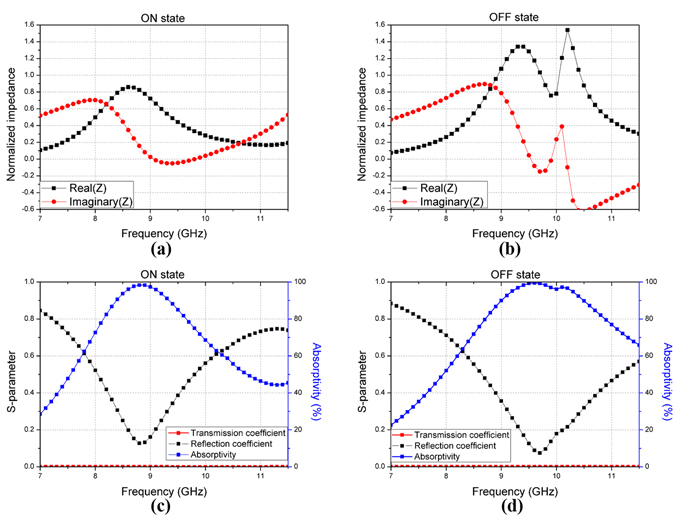
Normalized complex intrinsic impedance at (**a**) ON state, **(b)** OFF state, the simulated reflection and transmission coefficient and absorptivity of the proposed absorber at (**c**) ON state, **(d)** OFF state.

Figure 5(a) and (b) demonstrate the normalized complex intrinsic impedance (Z) at the top of the proposed absorber under normal incidence. The intrinsic complex impedance matrix (Z) can be calculated from the S-parameter (S) as follows (2)^21^:

2 $${\bf{Z}}=({\bf{U}}+{\bf{S}}){({\bf{U}}-{\bf{S}})}^{-1}.$$

where U is an identity matrix.

Normalized means that the calculated intrinsic complex impedance matrix is divided by the intrinsic impedance of free space (377 Ω). The frequency when the imaginary part value of the intrinsic impedance is 0, is the resonant frequency due to LC resonance. At this frequency, the real part value of the intrinsic impedance is closer to one, and the impedance matching between free space and proposed absorber is improved. Figure 5(c) and (d) show the simulated reflection and transmission coefficients and the absorptivity at each state. In this figure, even though there are via holes, the transmission wave is not in the proposed absorber.

**Figure 6:**
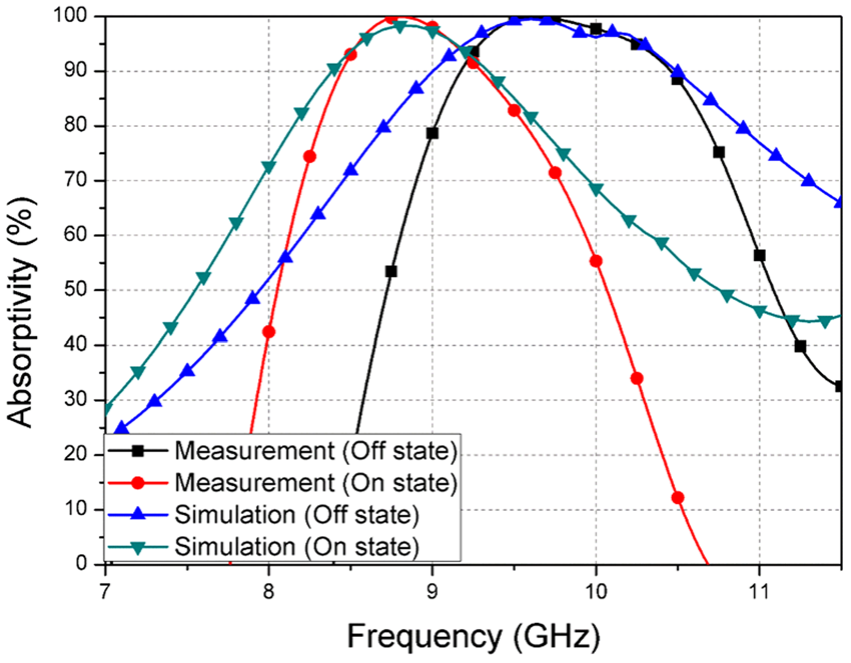
Measured and simulated absorptivity of the proposed absorber.

Figure 6 shows the measurement setup for the fabricated prototype. Wedge-tapered absorbers are placed around the prototype, to prevent signals reflected from the other parts. The reflection coefficient is measured using a horn gain antenna and an Anritsu MS2038C vector network analyzer (VNA). We used the WR-90 standard gain horn antenna with a norminal gain of 15 dB. Its 10-dB return loss bandwidth is 8.2–12.4 GHz. The absorptivity A(ω) of the proposed absorber is calculated using the measured reflection coefficient Γ(ω), as is shown the following equation (5):

3 $${\rm{A}}(\omega )=1-{\rm{\Gamma }}(\omega )-{\rm{T}}(\omega ),$$

where T(ω) is the transmission coefficient, but in this case, the transmission coefficient is zero because of fully covered with conductor on bottom layer.

**Figure 7:**
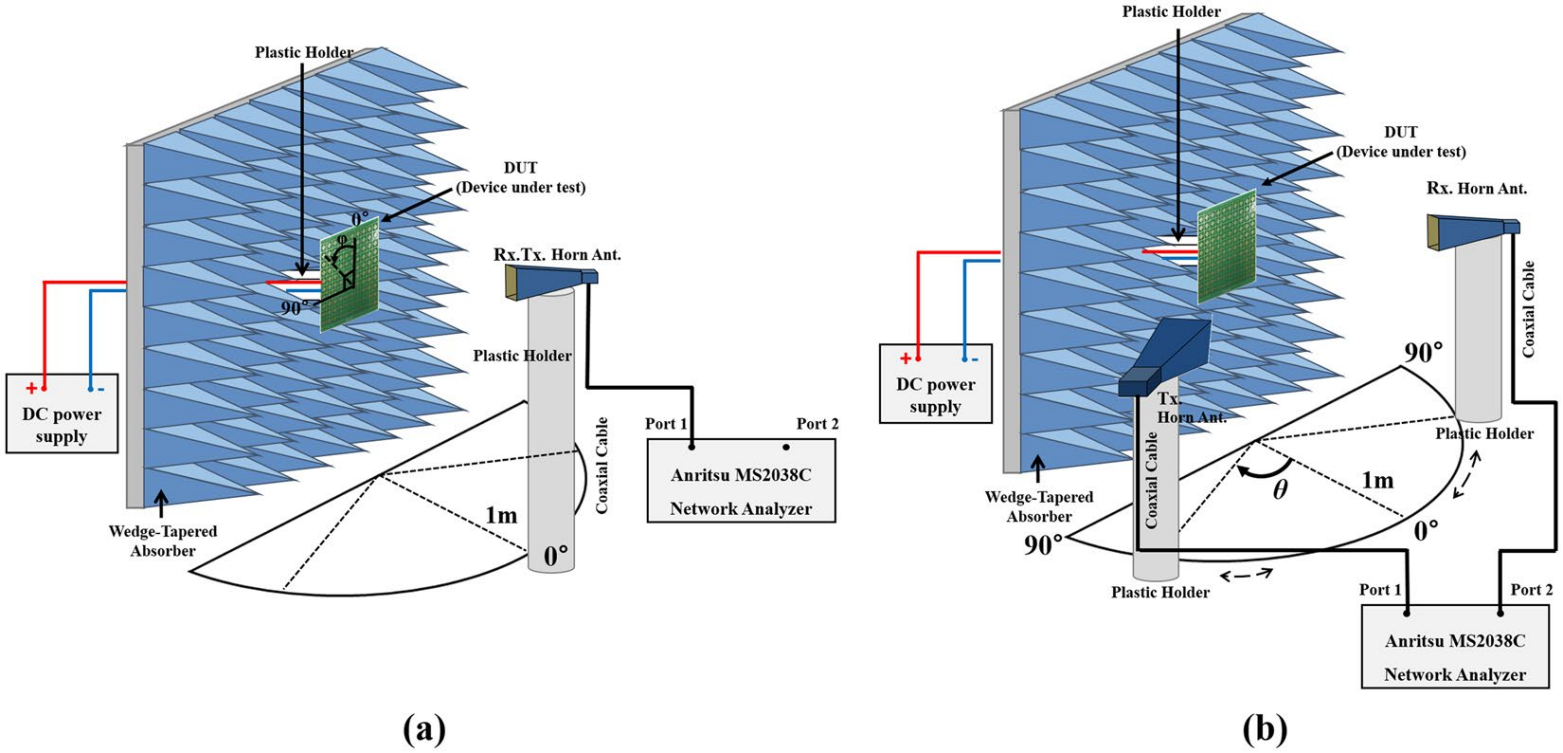
Measurement setup for different **(a)** polarization angles (φ) and **(b)** incident angles (θ).

Therefore, the absorptivity is calculated using only the measured reflection coefficient. To satisfy the far field condition, the horn gain antenna from the prototype sample, is located. Moreover, a time gating method is used to measure the just reflected wave from the prototype sample. A DC power supply is used to supply the PIN diode with power. Before measuring the prototype, the reflection coefficient of a conductor plate of the same size, is measured, to make reference |Γ| = 1^22^. The reflection coefficient of the prototype is the measured relative to the reference. Figure 7(a) shows the measurement results and simulation results under normal incidence. The proposed absorber has a 90% absorbing bandwidth from 8.45–9.3 GHz, when the PIN diode is in the ON state. When the PIN diode is in the OFF state, the 90% absorbing bandwidth is from 9.2–10.45 GHz. In Table 1, we compared the absorbing bandwidth of the proposed metamaterial absorber using the PIN diode, with the results of other researches. It is observed that the absorber proposed in this study, shows an absorption ratio and bandwidth that are similar to or better than those recorded in other researches.

**Table 1:** Comparison of the proposed metamaterial absorber using PIN diode with other researches. [unit: GHz].

Reference paper	ON state				OFF state			
	Absorbing BW	BW	Physical Size^1^ (mm^2^)	Electrical Size^2)^ (λ^2^)	Absorbing BW	BW	Physical Size (mm^2^)	Electrical Size (λ^2^)
23	12–12.8	0.8 (6.4%)	14 × 14	0.58 × 0.58	7.1–8.1	1 (13.2%)	14 × 14	0.58 × 0.58
24	4.75–4.85	0.1 (2%)	9 × 8.4	0.14 × 0.13	4.05–4.15	0.1 (2.4%)	9 × 8.4	0.14 × 0.13
15	2.85–3.15	0.3 (10%)	26 × 26	0.26 × 0.26	3.8–4.2	0.4 (10%)	26 × 26	0.26 × 0.26
25	2.45–2.55	0.1 (4%)	10.5 × 17.4	0.08 × 0.14	2.95–3.05	0.1 (3.3%)	10.5 × 17.4	0.08 × 0.14
Proposed work	8.4–9.3	0.9 (10.2%)	16 × 16	0.47 × 0.47	9.2–10.5	1.3 (13.2%)	16 × 16	0.52 × 0.52

^1)^Physical Size of the unit cell.

^2)^Electri cal Size is calcualted at the center frequency of the absorbing bandwidth.

**Figure 8:**
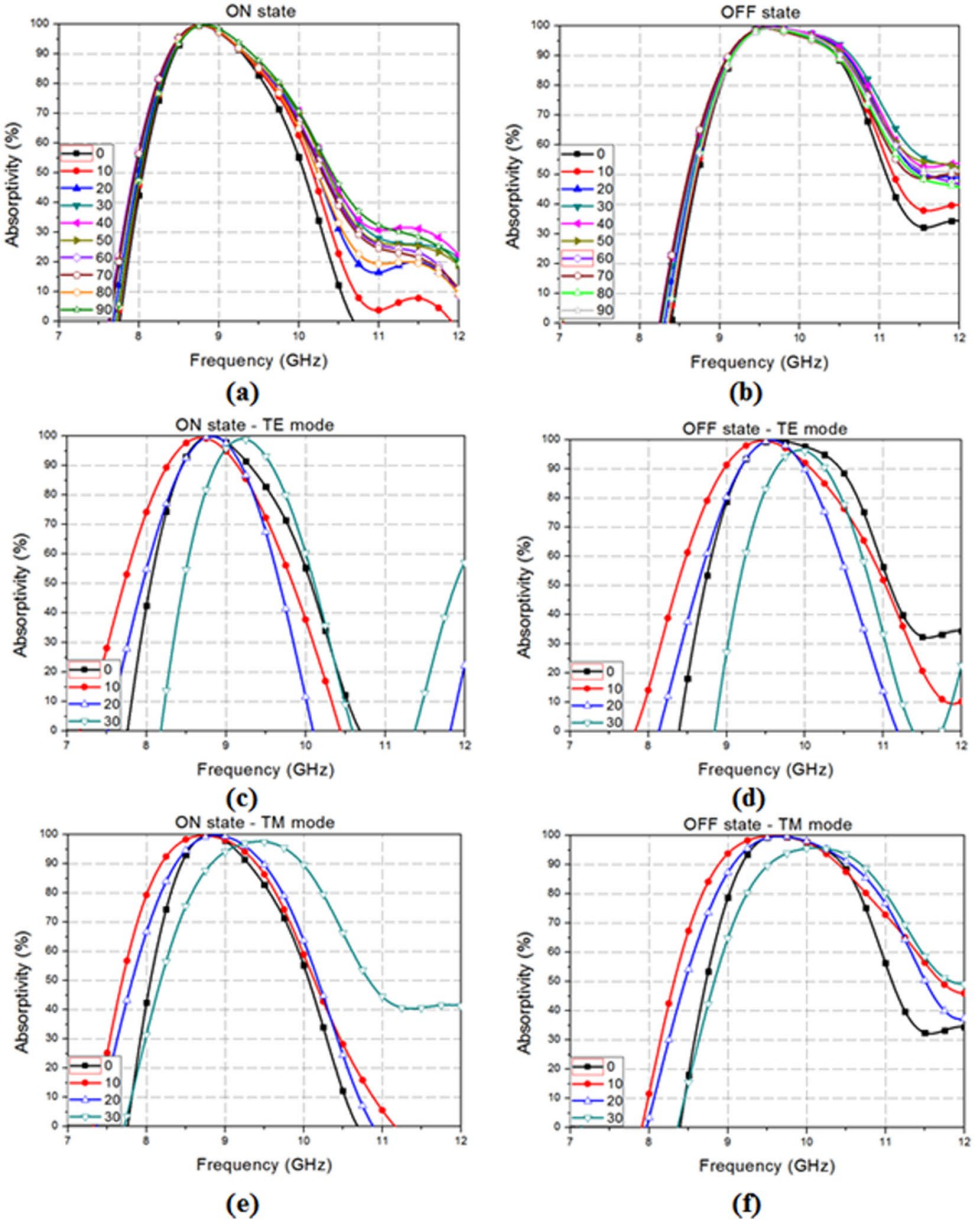
Measured absorptivity at different polarization angles (φ) at **(a)** ON state and **(b)** OFF state, at different incident angles (φ) for TE mode at **(c)** ON state and **(d)** OFF state, for TM mode at **(e)** ON state and **(f)** OFF state.

Additional measurements are performed to verify that the performance of the proposed absorber is independent of the polarization and incident angles. Figure 8(a) and (b) show the absorptivity at different polarization angles (φ). The measurement setup is shown in Fig. 7(a). The absorptivity is measured by changing the polarization angle (φ) from 0°–90° by rotating the prototype sample, while keeping all the other conditions the same. From the measurement results, the proposed absorber shows no change in performance with respect to the change of polarization of both the ON and OFF state. This is due to the unit cell being designed as a horizontally and vertically symmetrical structure, including the DC bias network, electronic devices, and conductor patterns. Consequently, the proposed absorber gives the advantage of not only having a switchable frequency band, but also an insensitivity to changes in the polarization angles.

Next, the measurement is done to demonstrate the performance of the absorber with respect to a changing incident angle (θ), as shown in Fig. 7(b). Unlike the previous polarization insensitivity experiment, two horn antennas are used to measure absorptivity. The two horn antennas are placed at the same angle from 0°–30° according to the incident angle (θ), to measure the reflected signal. In addition, the reflection coefficient is measured for the two polarizations, including the transverse electric (TE) mode and the transverse magnetic (TM) mode, because the reflection coefficient differs between the perpendicular (Γ_⊥_) and parallel polarization (Γ_||_), as shown by the following equation:

4 $${\rm{\Gamma }}{}_{\perp }(\omega )=\frac{{Z}_{M}(\omega )\cos \,{\theta }_{i}-{Z}_{0}\,\cos \,{\theta }_{t}}{{Z}_{M}(\omega )\cos \,{\theta }_{i}+{Z}_{0}\,\cos \,{\theta }_{t}}$$

5 $${\rm{\Gamma }}{}_{||}(\omega )=\frac{{Z}_{M}(\omega )\cos \,{\theta }_{t}-{Z}_{0}\,\cos \,{\theta }_{i}}{{Z}_{M}(\omega )\cos \,{\theta }_{t}+{Z}_{0}\,\cos \,{\theta }_{i}},$$

where *θ*_i_ and *θ*_t_ are incidence angle and transmitted angle, respectively.

Figure 8(c) and (d) show the absorptivity at different incident angles (θ) for the TE mode from 0°–30°. When PIN the diode is in the ON state, the absorptivity remains the same up to 20°. However, when the incident angle is 30°, the absorbing bandwidth is blue shifted around 0.5 GHz. Even when the PIN diode is in the OFF state, it exhibits the same result. Figure 8(e) and (f) show the absorptivity values at different incident angles (θ) for the TM mode from 0°–30°. Likewise, the measured results of the TE mode, irrespective of the PIN diode state, reveal that the absorptivity is maintained up to 20°, but it is also blue shifted around 0.5 GHz when the incident angle is 30°. The difference between the measurement results of the TE and the TM mode is the absorbing frequency bandwidth, which is wider in the TM mode than in the TE mode.

## Discussion

In this paper, the novel electronically switchable broadband metamaterial absorber, using PIN diode, is proposed. The proposed absorber is designed as a horizontally and vertically symmetrical structure, including the DC bias network, electronic devices, and conductor patterns in order to satisfy the condition of polarization insensitivity. To verify the performance of the proposed absorber, it is designed using a full wave simulation tool, and a fabricated prototype sample. The total size of the prototype is 162 × 162 mm, consisting of 10 × 10 unit cells. Conductor patterns are implemented using wet etching process. The electronic devices such as chip resistors, chip inductors, and PIN diodes are soldered onto the substrate, using surface mount technology (SMT). After fabricating the prototype sample, the experimental measurement setup is created to measure the performance of the proposed absorber with respect the change of the polarization (φ) and incident angles (θ). As a result, the proposed absorber has absorbing frequency bandwidths from 8.45–9.3 GHz and 9.2–10.45 GHz, when the PIN diode is switched ON and OFF state, respectively. The proposed absorber has polarization insensitivity, irrespective of the state of the PIN diode. The performance of the proposed absorber is maintained when the incident angle is varied from 0°–20°, irrespective of the state of the PIN diode, for both the TE and the TM modes. The proposed absorber has broadband and frequency switching characteristics that are similar to or better than those recorded in other researches using the PIN diode.

## Methods

### Measurement

Wedge-tapered absorbers are placed around the prototype, to prevent signals reflected from the other parts. The reflection coefficient is measured using horn gain antennas and an Anritsu MS2038C vector network analyzer (VNA). To satisfy the far field condition, the horn gain antenna from the prototype sample, is located. Moreover, a time gating method is used to measure the just reflected wave from the prototype sample. A DC power supply is used to supply the PIN diode with power. The fabricated metamaterial absorber consists of 400 PIN diodes. In order to turn on all diodes, 0.108 W is consumed with 9 DC-V. Before measuring the prototype, the reflection coefficient of a conductor plate of the same size, is measured, to make reference |Γ| = 1^22^. The reflection coefficient of the prototype is measured relative to the reference. Additional measurements are performed to verify that the performance of the proposed absorber is independent of the polarization and incident angles. The absorptivity is measured by changing the polarization angle (φ) from 0°–90° by rotating the prototype sample, while keeping all the other conditions the same. Next, the measurement is done to demonstrate the performance of the absorber with respect to a changing incident angle (θ). Unlike the previous polarization insensitivity experiment, two horn antennas are used to measure absorptivity. The two horn antennas are placed at the same angle from 0°–30° according to the incident angle (θ), to measure the reflected signal. In addition, the reflection coefficient is measured for the two polarizations, including the transverse electric (TE) mode and the transverse magnetic (TM) mode, because the reflection coefficient differs between the perpendicular (Γ_⊥_) and parallel polarization (Γ_||_).

### Simulation

In this study, the proposed absorber is designed using the ANSYS HFSS (high frequency structural simulator), which is a FEM (finite element method) based simulation tool. In the simulator, after only one unit cell is designed, the master and slave pair as a boundary condition are set up to assume an infinite periodic structure, where the E-field on one surface matches the E-field on another to within a phase difference. They force the E-field at each point on the slave boundary to match the E-field to within a phase difference at each corresponding point on the master boundary, to within a phase difference. In addition, the Floquet ports are used to excite the periodic structure^26^ in Fig. 3.
